# Interleukins 7 and 15 Maintain Human T Cell Proliferative Capacity through STAT5 Signaling

**DOI:** 10.1371/journal.pone.0166280

**Published:** 2016-11-17

**Authors:** Adam Drake, Mandeep Kaur, Bettina P. Iliopoulou, Ryan Phennicie, Amanda Hanson, Jianzhu Chen

**Affiliations:** Koch Institute for Integrative Cancer Research and Department of Biology, Massachusetts Institute of Technology, Cambridge, Massachusetts 02139, United States of America; Instituto Nacional de Ciencias Medicas y Nutricion Salvador Zubiran, MEXICO

## Abstract

T lymphocytes require signals from self-peptides and cytokines, most notably interleukins 7 and 15 (IL-7, IL-15), for survival. While mouse T cells die rapidly if IL-7 or IL-15 is withdrawn, human T cells can survive prolonged withdrawal of IL-7 and IL-15. Here we show that IL-7 and IL-15 are required to maintain human T cell proliferative capacity through the STAT5 signaling pathway. T cells from humanized mice proliferate better if stimulated in the presence of human IL-7 or IL-15 or if T cells are exposed to human IL-7 or IL-15 in mice. Freshly isolated T cells from human peripheral blood lose proliferative capacity if cultured for 24 hours in the absence of IL-7 or IL-15. We further show that phosphorylation of STAT5 correlates with proliferation and inhibition of STAT5 reduces proliferation. These results reveal a novel role of IL-7 and IL-15 in maintaining human T cell function, provide an explanation for T cell dysfunction in humanized mice, and have significant implications for *in vitro* studies with human T cells.

## Introduction

Following their development in the thymus, naïve T cells circulate in the lymphoid tissues where they survey peptides presented on the major histocompatibility complex (pMHC) for cognate antigens and to access survival signals. Under steady state conditions, survival of naïve T cells requires two signals: one from T cell receptor (TCR) engagement with self-pMHC and another from pro-survival cytokines such as interleukin (IL)-7 and IL-15. In the lymph node, pMHC complexes are usually presented by resident dendritic cells (DC) whereas IL-7 is secreted by stromal cells and IL-15 by DCs [1]. During periods of lymphopenia, the elevated levels of these survival signals can promote T cell proliferation to restore T cell numbers [1]. These roles of IL-7 and IL-15 have been defined by studies of mouse T cells, especially in knockout animals. However, human T cells exhibit significant differences to their murine counterparts [2,3]. For example, human and mouse T cells differ significantly in their dependence upon survival cytokines. Murine T cells require IL-7 to survive in culture and die rapidly without it [4]. Human T cells on the other hand can survive extended culture without any survival cytokine being provided [3].

Despite this significant functional difference in outcome further study of IL-7 and IL-15, their receptors and signaling pathways has shown that signaling is similar in both species. Binding of IL-7 or IL-15 to their respective receptors induces a series of signaling events involving phosphorylation of the common gamma chain (γc), Janus kinases, and signal transducer and activator of transcription 5 (STAT5), which eventually lead to change in gene transcription and biological effects, such as survival and proliferation. IL-7 and IL-15 are two members of a family of cytokines, consisting of IL2, IL4, IL-7, IL9, IL-15 and IL21, which all share γc as part of their receptors [5]. IL2, IL4, IL9 and IL21 are all viewed primarily as modulators of the immune response while IL-7 is seen as a primarily homeostatic cytokine and IL-15 is seen as fulfilling both roles due to the vital survival role this cytokine plays in T cells. As a result of the difficulty of separating survival and function in murine systems the functional role of these cytokines on T cells in healthy humans is unclear.

For ethical and practical reasons, the study of human T cells is usually carried out *in vitro* using T cells isolated from peripheral blood. To study human T cells and immune cells *in vivo*, one approach is to reconstitute the human immune system in mice. These humanized mice are constructed by engrafting CD34^+^ hematopoietic stem/progenitor cells (HSPCs) into immunodeficient mice, such as NOD-*SCID Il2r*γ^*-/-*^ (NSG) mice, which lack T, B and NK cells [6]. Development of the engrafted HSPCs leads to reconstitution of human immune cells, including T and B cells, in the recipient mice. Although a significant level of human T cells are usually generated in humanized mice, these T cells do not mount robust immune responses *in vivo* and activate inefficiently *in vitro* [7–10]. While human T cells respond to murine IL-7 and IL-15, and this response is sufficient for T cells to develop in humanized mice the mouse cytokines are not nearly as effective at stimulating the human receptors as their human counterparts. For example mouse IL-7 has been shown to have ~100x lower affinity for the human receptor than human IL-7 [11].

Various approaches, including provision of human cytokines, have been used to improve the functionality of human T cells in humanized mice [12]. Several groups have shown that providing human IL-7 or IL-15 generates superior immune responses in humanized mice [11,13–15]. These works focused on the positive effects of IL-7 and IL-15 on overall T cell numbers and demonstrated superior responses to *in vivo* stimulation. This was attributed to increased thymic output and homeostatic expansion of T cells resulting in greater numbers of cells and increased TCR diversity in the treated mice. However, little is known about the basal functional state of human T cells in humanized mice.

Here we have studied human T cells from humanized mice and compared them to T cells from human peripheral blood. Surprisingly, our results show that IL-7 or IL-15 is required to maintain optimal proliferation capacity of human T cells, but not survival. We further show that the effect of IL-7 and IL-15 is mediated through STAT5 signaling pathway. This novel role of IL-7 and IL-15 provides an explanation for the T cell dysfunction in humanized mice, has significant implications for *in vitro* studies with human T cells and potentially alters the interpretation of the underlying pathology of human diseases where IL-7 or IL-15 is dysregulated.

## Materials and Methods

### Ethics Statement

All research with human samples and mice was performed in compliance with the institutional guidelines, the World Medical Association’s Declaration of Helsinki and the US Department of Health and Human Services Guide for the Care and Use of Laboratory Animals. The Committee on Animal Care at Massachusetts Institute of Technology (MIT) reviewed and approved all of the studies described here. The MIT Committee on the Use of Humans as Experimental Subjects granted the research a waiver as all human samples (umbilical cord blood, adult peripheral blood, and fetal liver) were collected anonymously with informed consent by a third party and purchased from that party for the research.

### Mice

NOD-*SCID Il2r*γ^*-/-*^ mice were purchased from the Jackson Laboratories and bred in the animal facility at MIT. Humanized mice were constructed as reported previously [16]. Briefly, pups within 48 hrs. of birth were sub-lethally irradiated with 100 rads using a Gamma Cell 40 Cesium source, expanded or unexpanded HSPCs were transferred by intracardiac injection. Typically, ~100,000 CD34^+^CD133^+^ cells were transferred per animal. Around 12 weeks of age, human leukocyte reconstitution was determined by flow cytometry of peripheral blood mononuclear cells (PBMCs). Chimerism, or the level of human leukocyte reconstitution, was calculated as follows: Chimerism = %CD45^+^ human cell / (%CD45^+^ human cell + %CD45^+^ mouse cell). Mice with 20% or more human CD45-positive leukocytes and at least 30% T cells among human cells in PBMCs were used in the present study. Hydrodynamic injections used to express human IL-7 and IL-15 in mice were conducted as previously reported [17]. Mice were analyzed 7 days after injection.

### Tissue preparation, antibodies and flow cytometry

T cells were enriched from spleens using both mouse and human T cell negative selection kits from StemCell Technologies. Blood cells were labeled (based on cell number) with binding reagents (selection cocktail and biotin crosslinker) from both kits followed by a single dose of magnetic beads followed by manual separation. Purities of T cells were >80%. FITC, PE, PerCP/cy5.5, APC, PE/Cy7 or APC/Cy7 conjugated antibodies, including human CD3, CD4, CD5, CD8, CD25, CD27, CD28, CD34 CD45, CD69, TCRβ and murine CD45.1, were obtained from Biolegend. Anti-CD133 antibody was obtained from Miltenyi. Cells were stained with appropriate combination of antibodies and then analyzed on FACScalibur, FACS-Canto or LSR II flow cytometers (Beckton-Dickinson) or an AccuriC6 flow cytometer. Dead cells were excluded from analysis by DAPI or propidium iodide staining. T cells were gated on FSC/SSC, live cells, CD3^+^ then subdivided into CD4^+^ and CD8^+^ cells and assessed for markers as reported.

### Cell isolation and culture

Human CD34^+^ HSPCs were purified from fetal liver as previously described [18]. Alternatively, CD133^+^ cells were purified from fresh umbilical cord blood purchased from National Disease Research Interchange (NDRI) and cultured as previously described [19]. Human peripheral blood was obtained from Research Blood Components (www.researchbloodcomponents.com) of Boston, Massachusetts. Samples were collected with NaHep, shipped on ice, and processed immediately upon arrival, typically within 2–3 hours of blood collection. T cells were purified by Rosettesep granulocyte depletion (StemCell Technologies) according to the manufacturers guidelines, which involved discontinuous gradient centrifugation at 2000g for 40 mins without brake using (1.078 g/ml) Ficoll Paque Plus (GE healthcare); three washes with PBS, 0.2% BSA, 1mM EDTA followed by centrifugation 250g for 15 mins; RBC lysis (5 mins) using ACK buffer (Gibco); and human T cell negative enrichment (StemCell Technologies) according to the manufacturers guidelines. Typical T cell purities of >95% were obtained.

T cells were cultured in RPMI 1640 supplemented with 10% fetal bovine serum (Gibco), 50 μM β-mercaptoethanol (Gibco), L-glutamine (2 mM), penicillin and streptomycin (100 IU/ml, 100 μg/ml), sodium pyruvate (1 mM) and HEPES (10 mM) all from Corning as well as non-essential amino acids (100x, Lonza used at 1x). Cytokines IL2, IL4, IL-7, IL9, IL-15, IL21 TNFα and IFNγ (Peprotech) were used as indicated. T cells were stimulated with plate bound anti-CD3 and anti-CD28 antibodies (2 μg/ml) (Biolegend). STAT5 inhibitor (CAS 285986-31-4) was used at 50 μM.

### CFSE dilution assay

In order to assess cell division T cells were stained with CFSE (5 μM) (Molecular Probes) for 5 mins in serum free media and then washed with culture media once. Dilution of CFSE was assessed by flow cytometry based on unstimulated control cells.

### Western Blots

Purified T cells were lysed with RIPA buffer in the presence of protease inhibitors on ice. Cell lysates were then subjected to protein quantification with the Bradford reagent and equal amounts of protein was loaded on 10% polyacrylamide gels for gel electrophoresis followed by transfer onto nitrocellulose membranes. Transferred protein was blotted with the following antibodies from Cell Signaling: AKT (#9272), phospho-AKT Serine 473 (#9271), ERK1/2 (#9102), phospho-ERK1/2(#9101), STAT5 (#9363), phospho-STAT5 TYR694 (#9351), and GAPDH (#8884).

### Statistical analysis

Statistical analysis was carried out using Graphpad Prism 5. Two way ANOVA was used to compare series, paired two tailed T tests were used to compare T cells purified from individual donors and subjected to different treatments *in vitro*. Unpaired T tests were used to compare *in vivo* injections into littermates.

## Results

### T cells from humanized mice behave like cytokine-deprived human T cells

**Fig 1:**
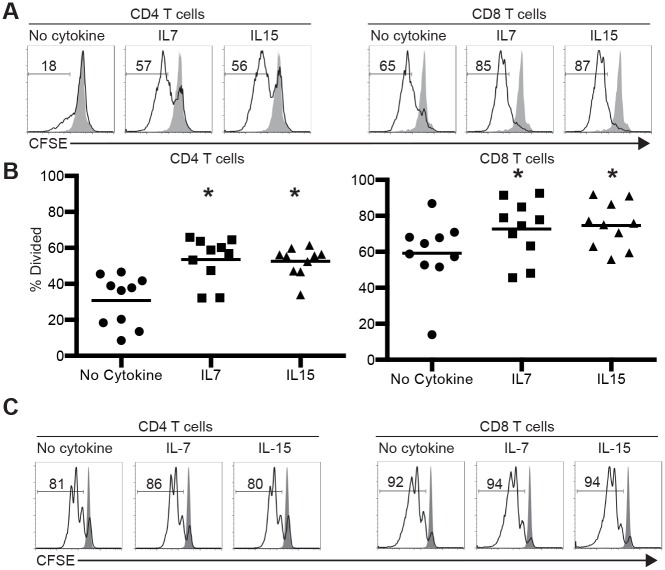
Proliferation of T cells from humanized mice and human peripheral blood with or without cytokines. Purified T cells (>95% CD3^+^) from humanized mice (A and B) or human peripheral blood (C) were labeled with CFSE and activated with plate-bound anti-CD3 and anti-CD28 for 3 days in the presence or absence of human IL-7 or IL-15. Cells were stained for CD4 and CD8 and analyzed by flow cytometry. Proliferation was assessed by CFSE dilution as compared to unstimulated purified T cells cultured without cytokines. (A) Representative CFSE histograms of stimulated (black line trace) and unstimulated (solid grey trace) CD4 or CD8 T cells from one humanized mouse. (B) Aggregated proliferation data from 10 mice that were constructed using 3 independent donor HSPCs. (C) Representative CFSE histograms of stimulated (black line trace) and unstimulated (solid grey trace) CD4 or CD8 T cells from one human peripheral blood sample. The numbers in A and C show percentages of cells that have diluted CFSE. * indicates P<0.05 by paired t test between no cytokine group and cytokine treated groups.

To investigate the molecular basis underlying the impaired function of human T cells from humanized mice, we compared T cell proliferation *in vitro* in the absence or presence of human IL-7 or IL-15. T cells were purified from the spleens of humanized mice between 12 to 20 weeks of age by negative selection, labeled with CFSE and stimulated with plate-bound anti-CD3 and anti-CD28 antibodies either in the presence or absence of human IL-7 or IL-15 (10ng/ml each). T cell proliferation as indicated by CFSE dilution was assessed 3 days later for CD4 and CD8 T cells separately. In the absence of cytokines, approximately 30% of CD4 T cells and ~60% of CD8 T cells diluted CFSE (Fig 1A and 1B). In the presence of IL-7 or IL-15, ~50% of CD4 T cells and ~75% of CD8 T cells diluted CFSE. Based on T cells from 10 humanized mice that were constructed using 3 independent donor CD34^+^ HSPCs, both CD4 and CD8 T cells proliferated significantly more in the presence of IL-7 or IL-15 than in the absence of these cytokines (Fig 1B).

We next determined whether IL-7 or IL-15 exerts a similar effect on freshly isolated human T cells from peripheral blood. Purified human T cells were labeled with CFSE and stimulated with plate-bound anti-CD3 and anti-CD28 in the presence or absence of IL-7 or IL-15 for 3 days. Approximately 80% of CD4 T cells and ~90% CD8 T cells diluted CFSE whether or not the cultures were supplemented with IL-7 or IL-15 (Fig 1C). Thus, unlike T cells from humanized mice, proliferation of freshly isolated human CD4 and CD8 T cells is not enhanced by the presence of IL-7 or IL-15 in the culture.

**Fig 2:**
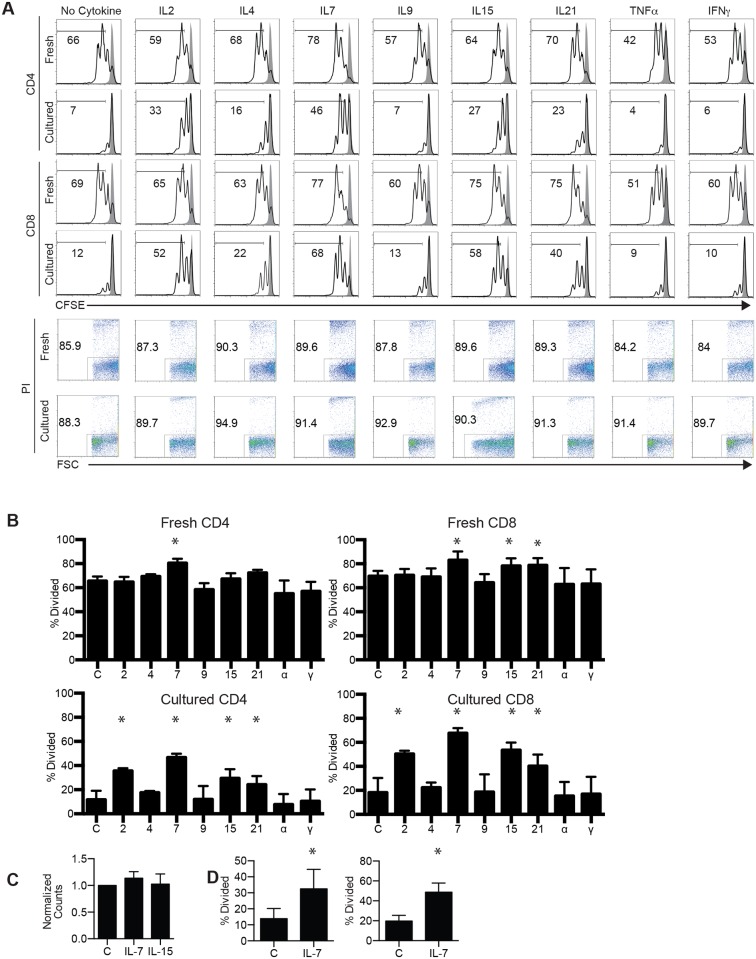
Effects of cytokines on T cell proliferation during activation of cytokine-deprived human T cells. Purified T cells from peripheral blood were cultured for 1 week in the absence of added cytokine. Both the cultured T cells and fresh purified T cells from the same donor were then labeled with CFSE, stimulated with plate-bound anti-CD3 and anti-CD28 for 3 days in the absence or presence of the indicated cytokines. Cells were stained for CD4 and CD8 and analyzed by flow cytometry. (A) Top and middle: Representative CFSE histograms of stimulated (black line trace) and unstimulated (solid grey trace) CD4 or CD8 T cells from one donor. Bottom: Viability of fresh and cultured cells following 3 days of stimulation. The numbers show percentages of cells that have diluted CFSE or in the gated region. (B) Aggregated data (mean ± SD) from 4 different donors. Cytokines are abbreviated to their corresponding number or alphabet and no cytokine is indicated by “C” for control. Cytokine concentrations used this experiment: IL2 (50U/ml), IL4 (100U/ml), IL-7 (10ng/ml), IL9 (50U/ml), IL-15 (10ng/ml), IL21 (10ng/ml), IFNγ (10ng/ml), and TNFα (10ng/ml). (C) Purified T cells from 3 donors were cultured for 1 week in the presence or absence of IL-7 (10ng/ml) or IL-15 (10ng/ml), live cells were then counted and cell counts normalized to cell number in the control cytokine-free condition, C indicates the control and IL-7 and IL-15 indicate culture with those cytokines. (D) CD4 and CD8 T cells from 3 donors were cultured for 24 hours without cytokine supplementation, labeled with CFSE and then activated with or without 10 ng/ml IL-7 for 3 days prior to analysis, C indicates control and IL-7 indicates treated cells. * indicates P<0.05 by paired T test between cytokine treatment to control without cytokines.

To test whether a similar proliferation defect occurs in human T cells we cultured purified cells for a week without cytokine supplementation. The cultured T cells and fresh T cells from the same donor were labeled with CFSE and stimulated with plate-bound anti-CD3 and anti-CD28 with the following cytokines: None (control), IL-2, IL-4, IL-7, IL9, IL-15, IL-21, TNFα or IFNγ for 3 days. Representative data are shown in Fig 2A and aggregated data in Fig 2B. When freshly isolated T cells were activated, similar percentages (60–70%) of CD4 and CD8 T cells diluted CFSE in the absence or presence of γc cytokines, although IL-7 consistently stimulated greater proliferation of both CD4 and CD8 T cells. Following culture for a week, the percentages of CD4 and CD8 T cells that diluted CFSE were reduced to ~10% for CD4 T cells and ~20% for CD8 T cells. The presence of IL-4, IL-9, TNFα and IFNγ did not significantly improve T cell proliferation. In contrast, human IL-2, IL-15, IL-21, and particularly IL-7 significantly restored CD4 and CD8 T cell proliferation as measured by CFSE dilution in live cells. As T cell death following cell division could contribute to the observed results, we assessed viability in both cultured and fresh T cells following stimulation. No significant difference was detected among all samples (Fig 2A bottom panels). Since, our observations could also be accounted for by cell death prior to activation, we cultured purified cells with or without IL-7 or IL-15 for 1 week and counted the viable cells. After 1 week of culture we recovered similar numbers of cells in all conditions as compared to the number of cells initially seeded on day 1 (Fig 2B), demonstrating no significant loss of viable cells in all conditions. Taken together these two observations indicate that cell death does not play a prominent role in differential proliferation either before or after activation.

Next we cultured freshly isolated human T cells from peripheral blood for 24 hours without cytokines and stimulated them with plate-bound anti-CD3 and anti-CD28 in the presence or absence of IL-7 for 3 days. Both CD4 and CD8 T cells in this shorter IL-7 deprivation had significantly reduced proliferation (Fig 2D), indicating that the effects of cytokine withdrawal could occur within much shorter time. As a 7-day culture isn’t necessary to observe these differences we used this shorter cytokine deprivation for the remainder of our studies. Taken together, these results suggest that the defective proliferation of human T cells from humanized mice and cytokine-deprived cultures is likely due to an acute lack of γc cytokines, especially IL-7.

### Cytokine-deprived T cells are impaired in CD25 upregulation

**Fig 3:**
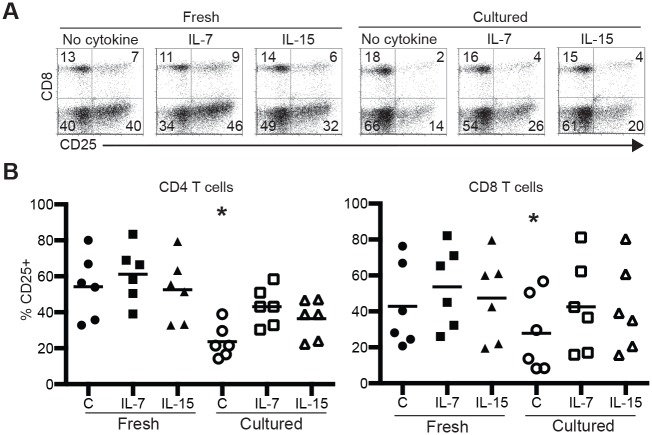
Impaired CD25 upregulation by cytokine-deprived T cells. Purified T cells from human peripheral blood were stimulated with plate-bound anti-CD3 and anti-CD28 for 2 days in the presence or absence of IL-7 or IL-15 or cultured for one day in the absence cytokine and then stimulated for 2 days. Cells were stained for CD3, CD4, CD8 and CD25 followed by flow cytometry. (A) Representative CD25 vs. CD8 staining profiles gating on CD3^+^ live cells. (B) Aggregate percentages of CD25^+^ CD4 and CD25^+^ CD8 T cells from 6 independent biological replicates. The numbers in A indicate the percentages of cells in the quadrants. * indicates P<0.05 by paired t test between no cytokine (C) cultured group and other groups.

We elected to focus on the effects of IL-7 and IL-15 as, based on mouse experiments, these cytokines are considered as primarily pro-survival while IL-2 is seen as pro-proliferation and the phenotype reported is thus expected for IL-2. In order to assess the effect of IL-7 and IL-15 on T cell activation prior to significant proliferation occurring around day three we assessed the expression of commonly used T cell activation markers CD69 and CD25 by flow cytometry. Freshly purified human T cells from peripheral blood were stimulated with plate-bound anti-CD3 and anti-CD28 in the absence or presence of IL-7 or IL-15, or cultured in the absence of cytokines for one day and then stimulated in the absence or presence of IL-7 or IL-15. Expression of CD69 and CD25 was measured one and two days after stimulation. Neither cell size (as measured by forward scatter) nor the expression level of CD69 was statistically different under various conditions (data not shown), although CD69 was generally lower on cultured T cells not exposed to IL-7 or IL-15. When cultured CD4 and CD8 T cells were stimulated without cytokines, the percentage of cells that upregulated CD25 was reduced by approximately 2-fold as compared to the corresponding fresh T cells (Fig 3, p<0.05). When cultured CD4 and CD8 T cells were stimulated in the presence of IL-7 or IL-15, the reduction in CD25^+^ T cells was either abolished (for CD8 T cells) or reduced to half (for CD4 T cells). Thus, deprivation of IL-7 or IL-15 impairs T cell activation.

### STAT5 signaling pathway mediates T cell proliferative capacity

We investigated the mechanism underlying the cytokine deprivation-induced changes in T cells. As changes in cell surface receptor expression would affect subsequent signaling we measured the levels of key receptors important for T cell activation, including CD3, CD4, CD5, CD8, CD27, CD28 and TCR. No significant difference was detected in any of these receptors among freshly isolated human T cells or the same human T cells that had been cultured in the absence or presence of IL-7 or IL-15 for 24 hours (data not shown).

**Fig 4:**
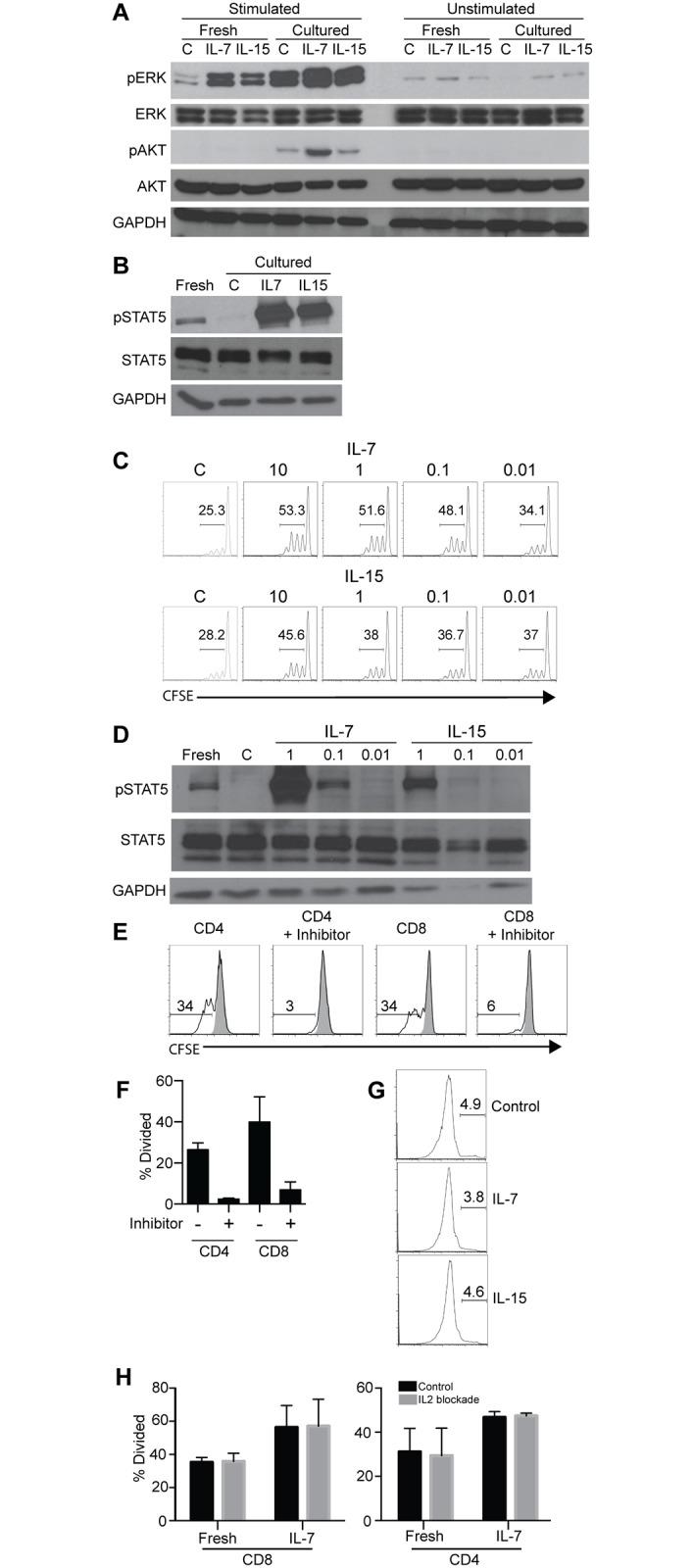
Impaired STAT5 phosphorylation in cytokine-deprived T cells. (A) Western blotting showing phosphorylated and total ERK and AKT in human T cells following various treatments. Freshly purified human T cells from peripheral blood were either unstimulated or stimulated with anti-CD3 and anti-CD28 for 5 minutes in the absence (-) or presence of IL-7 or IL-15 (10ng/ml each). Alternatively, purified human T cells were cultured for 24 hours in the absence or presence of IL-7 or IL-15 and then treated as above. Cells were lysed and total proteins were fractionated on a SDS-PAGE and then blotted for total and phosphorylated ERK and AKT. GAPDH was used a loading control. Representative data from 3 independent donors is shown. (B) Western blotting of phosphorylated and total STAT5 in freshly isolated and cultured human T cells. The culture conditions and replicates are the same as in A. (C) T cell proliferation in the presence of different concentrations of IL-7 or IL-15. Purified human T cells were cultured in the indicated concentrations (ng/ml) of IL-7 or IL-15, labeled with CFSE and activated with plate-bound anti-CD3 and anti-CD28 for 3 days, control cells provided with no cytokine are indicated with a C. Data shown is representative of biological duplicates used for Western blotting. (D) Western blotting of phosphorylated and total STAT5 with reduced concentrations of IL-7 and IL-15 (ng/ml). The experiment was performed as in B except the concentrations of IL-7 and IL-15 were reduced. Data shown is representative of biological duplicates. (E) Inhibition of human T cell proliferation by STAT5 inhibitor. Freshly purified human T cells were stimulated with plate-bound anti-CD3 and anti-CD28 in the absence or presence of STAT5 inhibitor. Shown are CFSE histograms of CD4 and CD8 T cells with (black line trace) or without (solid grey trace) stimulation. (F) Aggregate data of 4 biological replicates of STAT5 inhibitor treatment. (G) TUNEL stained purified T cells (>90%) were stained to assess for apoptotic DNA fragmentation. Data shown are representative flow cytometry plots of 3 independent biological replicates. (H) Comparison of proliferation of fresh and IL-7 treated (24h) cells in the absence or presence of IL-2 blocking antibody. T cells were activated with anti-CD3 and anti-CD28 for 3 days. Biological triplicates are shown.

Then we examined intracellular signaling looking for alterations in protein phosphorylation that correlated with the observed functional differences. We reasoned that the impaired proliferation of cytokine-deprived T cells is likely caused by the absence of an IL-7 or IL-15-induced signal. As ERK and AKT are downstream of both the γc cytokine receptor and the TCR, we assessed the phosphorylation levels of these two proteins [20,21]. Based on the impaired CD25 upregulation and proliferation we expected a reduced ERK and/or AKT signaling following culture in the absence of IL-7 or IL-15. Unexpectedly, following stimulation with anti-CD3 and anti-CD28, cultured human T cells, with or without cytokines, showed significantly higher levels of phosphorylation of ERK (pERK 1/2) and AKT (pAKT) at serine residue 473 (Fig 4A).

We considered STAT5, which is downstream of the γc and signals independently of TCR ligation [22]. Freshly isolated human T cells had a modest level of STAT5 phosphorylation (pSTAT5) (Fig 4B), which was lost following culture of T cells for 24 hours in the absence of cytokines. When T cells were cultured in the presence of either IL-7 or IL-15 the level of STAT5 phosphorylation was greatly enhanced. Because the far greater levels of pSTAT5 in T cells cultured with 10ng/ml of IL-7 or IL-15 did not correlate with corresponding levels of T cell proliferation, we tested the effects of serial dilutions of IL-7 and IL-15 on T cell proliferation and STAT5 phosphorylation. When T cells were cultured with 0.1ng/ml IL-7, i.e., 100-folder lower than the original concentration, an elevated proliferation was seen (Fig 4C). IL-15 was effective in sustaining increased T cell proliferation at 10 ng/ml, with reduced effectiveness at 1 ng/ml. We also compared the levels of pSTAT5 in cultured T cells at the same cytokine concentrations. At 0.1ng/ml IL-7 showed modest pSTAT5 phosphorylation, similar to that seen in freshly isolated T cells (Fig 4D). The level of pSTAT5 in cultured T cells at 1ng/ml IL-15 was similar to that seen in freshly isolated T cells while 0.1 ng/ml and below showed lower, though still detectable, levels of pSTAT5 (albeit with reduced loading at 1 ng/ml).

We further determined whether STAT5 phosphorylation is critical for T cell proliferation by using a specific STAT5 inhibitor CAS 285986-31-4 [23]. Freshly isolated human T cells were stimulated with plate-bound anti-CD3 and anti-CD28 in the absence or presence of the inhibitor. In the presence of the inhibitor, CD4 T cell proliferation was reduced by ~10x (26% vs. 2.5%) and CD8 T cell proliferation was reduced ~6x (40% vs. 7%) (Fig 4E and 4F). Thus, the effect of IL-7 and IL-15 in maintaining T cell proliferation is likely through STAT5 signaling.

IL-7 is known to promote survival through anti-apoptotic regulation of the BCL2 family [24]. To distinguish between reduced cell death and increased proliferation, we assessed apoptosis by intracellular TUNEL staining in T cells cultured with or without IL-7 or IL-15 for 24 hours and then stimulated with plate bound anti-CD3 and anti-CD28 for 3 days. Less than 5% TUNEL^+^ cells were detected under all conditions (Fig 4G), suggesting that differential apoptosis did not play a significant role in cytokine-enabled T cell proliferation.

We also considered indirect mechanisms, which might contribute to proliferation differences. As our assay is conducted following 1 day of culture then 3 days of stimulation with anti-CD3 and anti-CD28 antibodies there is time for T cells to produce autocrine or paracrine IL-2 to affect proliferation. In order to exclude this possibility, we assessed the effect of an IL-2 blocking antibody on the proliferation of cells activated in the presence of cytokine. We added anti-IL2 antibody (ab10752) throughout culture at 20 μg/ml [25]. We found that blocking IL-2 activity in culture had no significant effect on proliferation regardless of whether the cells were fresh or cultured with IL-7 (Fig 4H). We therefore concluded that IL-2 autocrine or paracrine signaling does not play a significant role in proliferation.

### Expression of IL-7 or IL-15 in humanized mice restores T cell proliferation capacity

**Fig 5:**
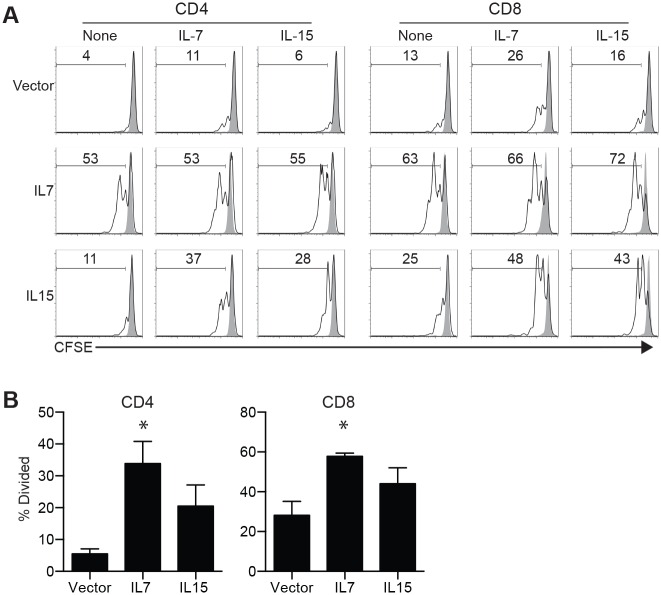
Rescue of T cell proliferation by IL-7 and IL-15 in humanized mice. Humanized mice were hydrodynamically injected with control plasmid DNA or plasmid encoding IL-7 or IL-15. Seven days later, T cells purified from the spleens of treated mice were activated with plate-bound anti-CD3 and anti-CD28 in the absence or presence of IL-7 or IL-15. (A) CFSE histograms from one vector injected mouse, one IL-7 plasmid injected mouse and one IL-15 plasmid injected mouse. (B) Aggregated data from four vector plasmid-injected mice, four IL-7 plasmid-injected mice and five IL-15 plasmid-injected mice. Each group contains mice generated from 3 independent HSPCs. * = P<0.05 in a paired T test between control and treated condition.

Given the body of literature documenting sub-optimal T cell function in humanized mice and our observations of increased proliferation and activation of human T cells in response to IL-7 and IL-15 *ex vivo* (Figs 1 to 4), we sought to test the role of human IL-7 and IL-15 in rescuing the defect of T cells in humanized mice *in vivo*. We expressed human IL-7 and IL-15 separately in humanized mice by hydrodynamic injection of cytokine-encoding plasmid [17]. Seven days following hydrodynamic injection no significant difference in the number of CD4 and CD8 T cells in the spleen was observed among the three groups (Table 1). Human T cells were purified, labeled with CFSE and activated with plate-bound anti-CD3 and anti-CD28 in the absence or presence of IL-7 or IL-15 for 3 days. As expected, T cells from empty vector injected mice did not proliferate much but increased proliferation was seen when activated in the presence of IL-7 or IL-15 *in vitro* (Fig 5A). In contrast, T cells from cytokine-treated mice showed more robust proliferation *in vitro* and reduced responsiveness to supplementation with IL-7 and IL-15 during *in vitro* stimulation. The increased proliferation of both CD4 and CD8 T cells from mice with IL-7 expression was significant (p<0.05) though not from mice with IL-15 expression (p>0.05) (Fig 5B). Thus, IL-7 and to a lesser extent IL-15 expression restores T cell proliferation capacity in humanized mice.

**Table 1:** Cell counts from mice used in Fig 5.

Mouse #	Group	Spleen Count	CD4+ %	CD8+ %	CD4 Count	CD8 count
275	Cntrl	2.80E+07	27%	12%	7.42E+06	3.47E+06
259	Cntrl	1.26E+07	15%	10%	1.94E+06	1.23E+06
14	Cntrl	4.00E+07	63%	29%	1.63E+07	7.58E+06
22	Cntrl	1.17E+08	61%	35%	5.39E+07	3.13E+07
15	IL-7	1.29E+08	61%	37%	5.89E+07	3.56E+07
46	IL-7	2.13E+08	66%	31%	1.17E+08	5.57E+07
256	IL-7	5.10E+07	15%	10%	7.85E+06	5.00E+06
257	IL-7	5.20E+07	29%	17%	1.49E+07	8.74E+06
260	IL-15	1.95E+07	25%	24%	4.78E+06	4.66E+06
261	IL-15	1.43E+07	13%	17%	1.90E+06	2.49E+06
263	IL-15	2.58E+07	14%	15%	3.69E+06	3.77E+06
16	IL-15	5.10E+07	50%	45%	1.47E+07	1.32E+07
9	IL-15	9.00E+07	54%	40%	4.05E+07	3.02E+07

## Discussion

In this study, we show that T cells from humanized mice proliferate better if stimulated in the presence of human IL-7 or IL-15 or if T cells are exposed to human IL-7 or IL-15 in mice. Freshly isolated human T cells from peripheral blood do not require IL-7 or IL-15 to proliferate optimally, but IL-7 or IL-15 is required to maintain T cell proliferative capacity during *in vitro* culture. The effect of IL-7 and IL-15 is likely mediated through STAT5 as IL-7 and IL-15 induce phosphorylation of STAT5 and inhibition of STAT5 results in suboptimal proliferation. These results highlight the important role of IL-7 and IL-15 in maintaining human T cell proliferative capacity, as opposed to the survival role seen in murine T cells. They also provide an explanation for T cell dysfunction in humanized mice, and have significant implications for *in vitro* studies with human T cells.

IL-7 and IL-15 are considered survival cytokines for T cells. This is especially true for naive T cells in mice, as they do not survive in the absence of IL-7, although survival of human T cells is less dependent on these cytokines. Here we show that IL-7 and IL-15 are critical for maintaining the optimal proliferative capacity of human T cells. Supporting this notion, human T cells from humanized mice have suboptimal proliferation in response to anti-CD3 and anti-CD28 stimulation. However, adding IL-7 or IL-15 to the culture or treating humanized mice with IL-7 or IL-15 can restore proliferation capacity. Suboptimal proliferation can also be induced in T cells from human peripheral blood by culturing them for 24 hours in the absence of IL-7 or IL-15. Furthermore, we show that the effect of IL-7 or IL-15 in maintaining T cell proliferative capacity is likely through STAT5 signaling. Binding of IL-7 and IL-15 to their receptors is known to stimulate STAT5 phosphorylation, which we show is correlated with T cell proliferation capacity. Inhibition of STAT5 abolishes T cell proliferation even in the presence of IL-7 or IL-15. Thus, despite extensive study of IL-7 and IL-15, our results reveal a so far unrecognized role of these survival cytokines in maintaining human T cell function.

Our findings have significant implications for the understanding of the common gamma chain cytokines, highlighting a common functional role for all these cytokines. IL-2, IL-4, IL-9 and IL-21 have been shown to play a significant role in promoting T cell function, particularly with regards to CD4 T cell polarization. In contrast, to date IL-7 and IL-15 are known to play a significant role in the development and survival of T cells. Our findings suggest additional functions of IL-7 and IL-15 in maintaining T cell proliferative capacity. Further, our work implicates STAT5, which is downstream of γc, as the mediator of this effect. The dominant STAT which signals downstream of γc receptor is STAT5 [26]. While STAT5 also signals downstream of IL-9, the lack of effect by this cytokine on T cell proliferation can be explained by the absence of IL9Rα expression on resting human T cells. The other less effective cytokines IL-4 and IL-21 primarily signal through STAT6 and STAT3 respectively, though both can also signal through STAT5 [26]. Our results are therefore consistent with the described signaling preferences of the various receptors.

Our findings also provide an explanation for the functional deficiency of human T cells generated in humanized mice. Engraftment of CD34^+^ HSPCs into immunodeficient mice leads to robust development of human T and B cells in the recipients. However, the human T cells are known to be functionally deficient, notably in their ability to proliferate *in vitro* [7]. Consistent with these previous observations, we show that human T cells from humanized mice had suboptimal proliferation in response to plate-bound anti-CD3 and anti-CD28 stimulation. More importantly, we show that the proliferative defect can be corrected by including IL-7 or IL-15 in the culture or by expressing IL-7 or IL-15 in humanized mice prior to harvesting the T cells for analysis. Our results are consistent with previous reports showing that human IL-7 and IL-15 increases TCR diversity and improve the overall T cell number in humanized mice [11,13,15]. Stromal cells and other non-hematopoietic lineage cells are the primary source of IL-7 and therefore humanized mice are deficient in human IL-7. IL-15 is produced by mononuclear phagocytes and retained on the cell surface through interaction with IL-15Rα chain. Due to the poor reconstitution of human myeloid cells in humanized mice, humanized mice are likely also deficient in human IL-15. In support of this notion, the level of human IL-7 and IL-15 is below detection by ELISA in the sera of humanized mice (unpublished data). Furthermore, although mouse IL-7 binds to the human IL-7 receptor, its affinity is ~100x lower compared to binding of human IL-7 to the human receptor [11]. Therefore, compared to normal humans, humanized mice probably maintain T cells in an IL-7 and IL-15 deficient environment. A great deal of work has been devoted to improving the reconstitution of the myeloid compartment by transgenically expressing human cytokines [27–33] and enhancing T cell function by improving thymic selection or expressing human MHC transgenes in mice [34–37]. Our study suggests that physiological expression of human IL-7 and IL-15 would substantially improve human T cell responses in humanized mice.

Our findings have significant implications for how *in vitro* studies with human T cells should be designed and interpreted. T cells from peripheral blood are widely used in studies of human T cell responses in both basic and clinical settings. Upregulation of activation markers and proliferation are key parameters that are usually assayed. We show that although freshly isolated human peripheral blood T cells respond robustly to anti-CD3 and anti-CD28 stimulation, their proliferation and upregulation of CD25 is attenuated once the cells are deprived of IL-7 or IL-15 for 24 hrs. Based on our findings, prolonged isolation procedures and culture prior to the actual assays should be avoided. If prolonged culture is unavoidable, inclusion of a common gamma chain cytokine such as IL-7 or IL-15 in the culture will help to maintain robust T cell proliferative responses. Ideally, the *in vitro* assays should include two controls—fresh untreated donor T cells assayed immediately and mock or vehicle treated T cells assayed at the end of the study. The first control gives the normal baseline and the second shows what effect (if any) assay incubation time has, making it possible to distinguish between effects that reverse inhibition and those that enhance T cell function.

The importance of our findings to *in vitro* studies is further illustrated by comparing our results with those from very similar experiments reported by Deshpande and colleagues recently [38]. In a series of experiments focusing on the role of IL-7 and IL-15 in rheumatoid arthritis (RA), Deshpande et al. reported that IL-7 and IL-15 cause increased sensitivity to ERK signaling and allow T cells to respond to self-antigens relevant to RA. Their results seem at first to directly contradict our findings, however the results from the two studies are consistent, as all of the T cells they assessed have been cultured for 24 hours prior to each assay. The data presented by Deshpande et al. are internally consistent, and their interpretation is logical. However, in light of our findings it is likely that IL-7 and IL-15 are restoring function in T cells rather than enhancing immune activation in many of their assays. We predict this awareness will improve the quality and reproducibility of data and that careful analysis of the methods in superficially contradictory studies may resolve apparent inconsistencies in the literature.

Finally, our results suggest an important spatial role for IL-7 and IL-15, and the γc cytokines more broadly in regulating peripheral immune responses. Our findings show that the functional effect of IL-7 and IL-15 is one of licensing full function rather than causing pathogenic gain of function. Where IL-7 and IL-15 have been implicated in disease pathology, elevated levels of cytokine may be licensing cells in inappropriate non-lymphoid locations, leading to autoimmune pathology. Work in a mouse lupus model by Gonzalez-Quintial and colleagues supports this hypothesis. They showed that excess IL-7, which is not consumed *in situ* by T cells, can lead to autoimmune pathology that can be treated by IL-7 signal blockade [39]. Further study of spatial deregulation of IL-7 production rather than overexpression and an explicit comparison of disease phenotypes (if any) would be of great interest. This hypothesis, if proven, would greatly strengthen the case for localized therapy to control disease.
